# High Plasma Exposure of Statins Associated With Increased Risk of Contrast-Induced Acute Kidney Injury in Chinese Patients With Coronary Artery Disease

**DOI:** 10.3389/fphar.2018.00427

**Published:** 2018-04-30

**Authors:** Liyun Cai, Xue Bai, Heping Lei, Hong Wu, Yong Liu, Qian Zhu, Shanshan Zhang, Yibin Liu, Qiuxiong Lin, Jiyan Chen, Bin Zhang, Guodong He, Qingshan Geng, Min Huang, Shilong Zhong

**Affiliations:** ^1^Guangdong Provincial Key Laboratory of Coronary Heart Disease Prevention, Guangdong Cardiovascular Institute, Guangdong General Hospital, Guangdong Academy of Medical Sciences, Guangzhou, China; ^2^School of Medicine, South China University of Technology, Guangzhou, China; ^3^Laboratory of Drug Metabolism and Pharmacokinetics, School of Pharmaceutical Sciences, Sun Yat-sen University, Guangzhou, China; ^4^Sun Yat-sen Memorial Hospital, Sun Yat-sen University, Guangzhou, China

**Keywords:** contrast-induced acute kidney injury, coronary angiography, atorvastatin, rosuvastatin, plasma exposure

## Abstract

The role of statins in reducing the incidence of contrast-induced acute kidney injury (CI-AKI) remains controversial. We sought to evaluate the association between CI-AKI and high plasma exposure of statins in coronary artery disease (CAD) patients undergoing coronary angiography (CAG). This association was first evaluated in 1,219 patients with CAD receiving atorvastatin (AT) therapy and validated in 635 patients receiving rosuvastatin (RST) therapy. The plasma concentrations of statins were quantified using validated UPLC-MS/MS methods and CI-AKI incidence was assessed during the first 48 h postoperatively. Among all participants (*n* = 1,854), AKI occurred in 57 of 1219 (4.7%) in the AT cohort and 30 of 635 (4.7%) in the RST cohort. High plasma AT-all exposure was associated with increased risk of CI-AKI (odds ratio [OR]: 2.265; 95% confidence interval [CI]: 1.609–3.187; *p* < 0.0001). Plasma AT-all concentration in the CI-AKI group (22.40 ± 24.63 ng/mL) was 2.6-fold higher than that in the control group (8.60 ± 9.65 ng/mL). High plasma RST exposure also significantly increased the risk of CI-AKI (OR: 2.281; 95% CI: 1.441–3.612; *p* = 0.0004). We further divided patients into two subgroups for each statin according to baseline renal function, and association between high plasma statin exposure and CI-AKI still remained highly significant in both subgroups. This study suggests for the first time that high plasma exposure of statins may significantly increase the risk of CI-AKI. Statins should be used with greater caution in CAD patients undergoing CAG to reduce the occurrence of CI-AKI.

## Introduction

Contrast-induced acute kidney injury (CI-AKI) is a known complication of intravascular administration of contrast media used in coronary angiography (CAG) and percutaneous coronary interventions (PCI) (Chalikias et al., 2016); and is associated with increased mortality, morbidity, healthcare expenditure, and prolonged hospital stay (Nash et al., 2002; Prasad et al., 2016). CI-AKI has become the third leading cause of iatrogenic renal failure in the United States (Prasad et al., 2016). Previous report indicated that even mild postoperative AKI is independently associated with an almost 5-fold increase in in-hospital death (Birnie et al., 2014). Clinically, the incidence of CI-AKI is greater in patients with cardiovascular diseases or pre-existing renal insufficiency (Goldenberg and Matetzky, 2005; Itoh et al., 2005; Ledneva et al., 2009). The reported incidence ranges from 2 to 50% (Aurelio and Durante, 2014).

Hydroxy-methylglutaryl-coenzyme A (HMG-CoA) reductase inhibitors (statins) are potent inhibitors of cholesterol biosynthesis and exert beneficial effects in the primary and secondary prevention of coronary artery disease (CAD). The prophylactic benefit of statins in reducing the incidence of CI-AKI has been investigated in several observational (Khanal et al., 2005; Patti et al., 2008; Lev et al., 2009) and randomized studies (Patti et al., 2011); however, other studies have reported inconsistent and conflicting results (Argalious et al., 2010; Mithani et al., 2011; Billings et al., 2016; Park et al., 2016). Therefore, whether preoperative statin therapy has a preventive, neutral, or detrimental role on AKI remains unclear and hotly debated. To the best of our knowledge, no studies have evaluated the relationship between high plasma exposure of statins and the risk of CI-AKI.

Therefore, the objective of the current study was to systematically investigate the effects of high plasma exposure of widely prescribed statins (atorvastatin [AT] and rosuvastatin [RST]) and their metabolites on the incidence of CI-AKI in patients with CAD undergoing CAG.

## Methods

### Ethics statement

The present study was approved by the Medical Ethical Review Committee of Guangdong General Hospital and conducted according to the Declaration of Helsinki. All participants gave written informed consent in accordance with the Declaration of Helsinki.

### Study design and patients

We conducted a prospective two-stage study to evaluate the effects of two statins on CI-AKI separately. In stage I (test set), 1,219 patients taking AT were recruited, including 1,023 patients without chronic kidney disease (CKD) and 196 patients with CKD. In stage II (validation set), 635 patients taking RST were enrolled for further validation; 531 of these were without CKD, whereas 104 were with CKD.

All patients were sequentially recruited in Guangdong General Hospital from January 2010 to December 2013 according to the same inclusion and exclusion criteria. Baseline information, including demographics, medical history, biochemical measurements, and medication was obtained from the hospital information database. Patients who underwent CAG and were diagnosed with CAD were included in the study.

The exclusion criteria included the followings: (1) age < 18 years or age > 80 years; (2) renal transplantation or dialysis; (3) liver insufficiency (defined as serum transaminase concentrations > 3 times the upper limit of normal [120 U/L], or a diagnosis of cirrhosis); (4) being pregnant or lactating; (5) advanced cancer or haemodialysis; (6) the concentrations of statins or their metabolites were lower than limit of detection (3:1 noise).

### Coronary angiography procedure

CAG is performed to define the extent and severity of CAD in patients with suspected symptoms whose clinical characteristics and results of non-invasive testing indicate a high likelihood of CAD and who are amenable to, and candidates for, coronary revascularization (Fihn et al., 2014). Information derived from the CAG procedure will be took into consideration in patient management, and the risks and benefits of the procedure have been carefully considered and understood by the patients.

We used the Synergy between PCI with TAXUS and Cardiac Surgery (SYNTAX) score, an angiographic scoring system to determine the complexity, severity, and atherosclerotic burden of CAD (Sianos et al., 2005; Ikeda et al., 2012). SYNTAX score has been shown to independently predict MACE and long-term prognosis risks in stable CAD patients who underwent revascularization (Serruys et al., 2009; Mohr et al., 2013).

Images of coronary angiograms were obtained with Syngo Dynamics cardiovascular imaging software (Siemens Medical Solutions USA, Inc., Malvern, Pennsylvania). The SYNTAX score was calculated for each patient using a computer program consisting of sequential and interactive self-guided questions according to the SYNTAX score calculator version 2.11. The SYNTAX score reflects a comprehensive anatomical assessment, and a low SYNTAX score was defined as ≤ 22, an intermediate score as 23 to 32, and a high score as ≥33.

### Clinical endpoint

The study endpoint was the diagnosis of postoperative CI-AKI. According to the Acute Kidney Injury Network criteria (Mehta et al., 2007), CI-AKI was diagnosed if a patient had an absolute increase in serum creatinine (sCr) concentration ≥0.3 mg/dL (26.4 μmol/L) from baseline or a relative increase ≥50% (1.5-fold from baseline) in sCr concentration for more than 6 h within 48 h after surgery.

CKD was defined as an estimated glomerular filtration rate (eGFR) < 60 mL/min per 1.73 m^2^ using the Modification of Diet in Renal Disease equation (National Kidney Foundation, 2002). SCr levels were measured upon admission and within 48 h after surgery. Alanine aminotransferase (ALT), aspartate aminotransferase (AST), cholesterol, creatine kinase (CK), creatine kinase MB (CKMB), and other standard clinical parameters were measured in the morning before the procedure.

### Plasma sample preparation

Each eligible patients had been taking the same dose of AT or RST for at least 7 days prior to blood sampling. Statin dosage was prescribed by physician in accordance with patients' condition. Blood samples were obtained at 10–12 h post-dose in the morning before the CAG and collected in EDTA-coated tubes. Plasma was separated within 2 h by centrifugation at 3,000 rpm for 10 min at 4°C and then stored at −80°C until analysis.

### Quantification of plasma concentrations of statins and their metabolites

A reliable assay of ultra-performance liquid chromatography coupled with tandem mass spectrometry (UPLC–MS/MS) was developed and validated for the quantification of AT, its five metabolites, and internal standard (IS) carbamazepine in human plasma as described previously (Cai et al., 2017).

A sensitive UPLC–MS/MS assay was also developed and validated for the simultaneous quantification of RST, rosuvastatin lactone (RSTL), and N-desmethyl rosuvastatin (DM-RST) in human plasma. All the three analytes and the corresponding IS (carbamazepine) were extracted from 200 μL buffered human plasma (adding 100 μL ammonium acetate of pH = 4.0 to 100 μL human plasma) by liquid–liquid extraction with ethyl acetate and then separated on an ACQUTY UPLC HSS T3 column (3.0 × 100 mm, 1.8 μm). The elution was performed at a rate of 0.3 mL/min using a mobile phase containing acetonitrile and 0.05% formic acid in water over a linear gradient of 30–85% acetonitrile. Mass detection was performed on a Waters Xevo TQ-S triple-quadrupole mass spectrometer in positive electrospray ionization mode. The responses of RST, RSTL, and DM-RST were optimized at the m/z 482.1→ 258.1, m/z464.1→ 270.1, m/z 468.0→ 258.0, respectively.

### Statistical analysis

The demographic and clinical characteristics were summarized using counts (percentages) for the categorical variables and mean (standard deviation, *SD*) for the continuous variables. As the ranges of the concentrations of statins and metabolites were skewed, logarithmic transformation was performed prior to analysis. AT-all is calculated amount of plasma AT concentration and equivalent concentrations of its two pharmacologically equipotent metabolites; this value represents overall therapeutic efficacy (Lennernas, 2003). Given that approximate 90% of plasma pharmacologically activity is accounted for RST, we only applied plasma RST concentration to represent the overall therapeutic efficacy (White, 2002).

Linear regression analysis was applied to evaluate the effects of the baseline demographic and clinical characteristics on the plasma concentrations of statins and metabolites. A univariate logistic regression analysis was conducted to evaluate the effects of plasma concentrations, baseline demographic, and clinical characteristics on the risk to CI-AKI and to calculate odds ratio (OR) and 95% confidence interval (CI). Variables with *p* < 0.05 were entered into the multivariate model, and only variables with *p* < 0.05 were retained in the model. *P* < 0.05 was considered statistically significant. Data analysis was performed using SAS 9.4 (SAS Inst, Cary, NC, USA).

### Predictive diagnostic power of variables for CI-AKI

In the study, the Daim package in R (version 3.2.3, http://www.R-project.org/) was used to construct the classification models. For each predictor variable, the true positive rate and false positive rate as a predictor of CI-AKI was evaluated by the receiver operating characteristic (ROC) curves using the area under the curve (AUC) as a measure of diagnostic effectiveness (Zweig and Campbell, 1993). First, every independent variable associated with CI-AKI were selected to construct the classifier for estimating the diagnostic effectiveness of a single predictor. Then, all significant variables were combined as a classifier for estimating the diagnostic effectiveness of variable combinations. The optimal cutoffs were calculated by selecting the data point that maximized the true positive rate and minimized the false positive rate.

## Results

### Patient characteristics and their effects on plasma statins and metabolites exposure

**Figure 1:**
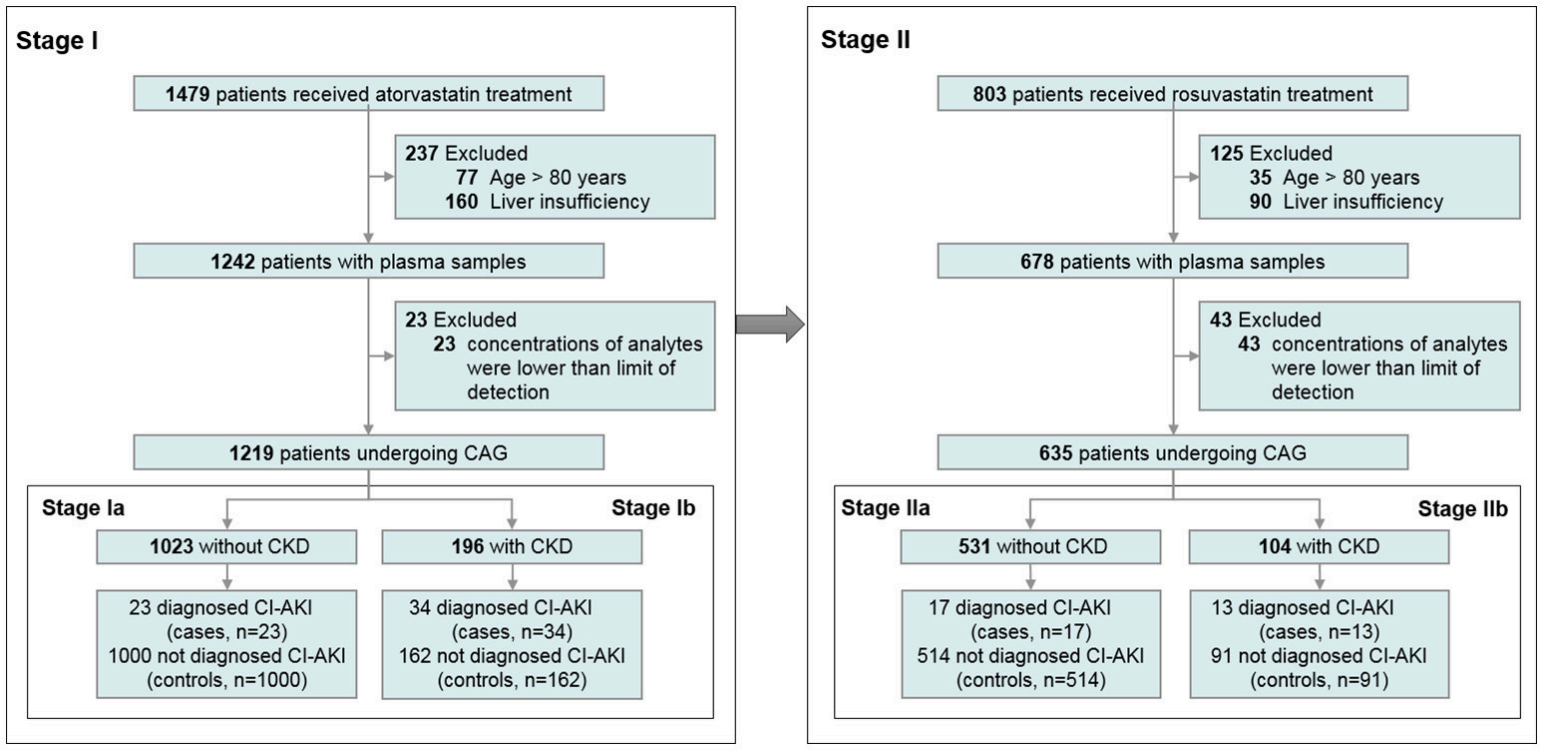
Flow chart of the enrolment of the participants. CAG, coronary angiography; CI-AKI, contrast-induced acute kidney injury; CKD, chronic kidney disease.

An overview of the enrolment of the patients is presented in Figure 1. In stage I, plasma concentrations of AT and its metabolites widely varied, which is consistent with published data (DeGorter et al., 2013). The concentrations of five metabolites were highly correlated with AT concentration (all *r* > 0.5, *p* < 0.0001). Among 1,219 patients with AT therapy, 21 (1.72%) were taking 10 mg AT, 1058 (86.79%) were 20 mg AT, and 140 (11.48%) were 40 mg AT compliance with prescription, respectively. Patients' baseline characteristics and their impacts on the AT concentration are summarized in Table 1.

**Table 1:** Patient characteristics and their effects on plasma concentration of AT-all.

**Characteristics**		**Value *N* (%) or mean ±*SD***	**Plasma AT-all concentration, ng/mL**				
				**Univariate analysis**		**Multivariate analysis**	
			**mean ±*SD***	**Estimate**	***p*-value**	**Estimate**	***p*-value**
**DEMOGRAPHIC DATA**							
Total number		1219	9.25 ± 11.19				
Age (years)		62.95 ± 10.00		0.0122	<0.0001	0.0140	<0.0001
Sex	Female	297 (24.36)	9.89 ± 14.40	−0.0032	0.9633		
	Male	922 (75.64)	9.04 ± 9.94				
Dosage (mg)	10	21 (1.72)	4.23 ± 3.69	0.0197	<0.0001	0.0173	<0.0001
	20	1058 (86.79)	8.72 ± 10.47				
	40	140 (11.48)	13.98 ± 15.27				
SYNTAX score		13.81 ± 12.19		0.0094	0.0001	0.0059	0.0161
**MEDICAL HISTORY**							
Arrhythmia	No	1104 (90.86)	9.08 ± 10.61	0.0707	0.4914		
	Yes	111 (9.14)	10.98 ± 15.86				
Diabetes	No	895 (73.66)	8.99 ± 10.56	0.0266	0.6924		
	Yes	320 (26.34)	9.99 ± 12.82				
Heart failure	No	1097 (90.29)	8.93 ± 10.46	0.1210	0.2261		
	Yes	118 (9.71)	12.24 ± 16.35				
Hypertension	No	499 (41.04)	8.40 ± 9.14	0.0881	0.1435		
	Yes	717 (58.96)	9.83 ± 12.41				
Hyperlipidemia	No	1073 (88.24)	9.36 ± 11.28	−0.0265	0.7734		
	Yes	143 (11.76)	8.40 ± 10.57				
**BIOCHEMICAL MEASUREMENTS**							
ALT, U/L		28.69 ± 16.80		0.0055	0.0017	0.0059	0.0012
AST, U/L		31.05 ± 30.42		0.0031	0.0014		
Scr, μmol/L		96.70 ± 81.69		0.0014	<0.0001	0.0012	0.0008
eGFR, mL/min/1.73 m^2^		91.26 ± 72.98		−0.0013	0.0013		
CK, U/L		158.91 ± 408.68		0.0001	0.4328		
CKMB, U/L		8.82 ± 13.67		0.0026	0.2603		
CHOL, mmol/L		4.32 ± 1.13		0.0318	0.2305		
LDLC, mmol/L		2.60 ± 0.94		0.0569	0.0739		
HDLC, mmol/L		0.99 ± 0.26		−0.1445	0.2058		
TRIG, mmol/L		1.58 ± 1.11		−0.0018	0.9463		
GLUC, mmol/L		6.66 ± 2.59		0.0267	0.0204		
Lpa, mg/L		295.05 ± 315.79		0.0003	0.0114		
APOA, g/L		1.06 ± 0.29		−0.3237	0.0048		
**MEDICATION**							
β-blockers	No	137 (11.27)	8.20 ± 8.00	0.0263	0.7787		
	Yes	1079 (88.73)	9.38 ± 11.54				
ACEIs	No	467 (38.4)	9.46 ± 11.17	−0.0255	0.6761		
	Yes	749 (61.6)	9.11 ± 11.22				
CCBs	No	848 (69.74)	8.93 ± 10.17	−0.0102	0.8743		
	Yes	368 (30.26)	9.97 ± 13.25				
PPIs	No	588 (48.36)	8.69 ± 10.79	0.0550	0.3535		
	Yes	628 (51.64)	9.77 ± 11.55				

*Estimates were calculated by applying a linear regression model.Variables with P < 0.05 were entered into the multivariate model, and only variables with P < 0.05 were retained in the model ACEIs, angiotensin converting enzyme inhibitors; ALT, alanine aminotransferase; APOA, apolipoprotein a; AST, aspartate aminotransferase; CCBs, calcium channel blockers; CHOL, cholesterol; CK, creatine kinase; CKMB, creatine kinase MB; eGFR, estimated glomerular filtration rate; GLUC, glucose; HDLC, high-density lipoprotein cholesterol; LDLC, low-density lipoprotein cholesterol; Lpa, lipoprotein (a); PPIs, proton pump inhibitors; Scr, serum creatinine; SD, standard deviation; SYNTAX score, Synergy between percutaneous coronary intervention with TAXUS and Cardiac Surgery score; TRIG, triglyceride*.

Univariate linear regression analysis showed that plasma AT-all concentration was affected by age, dosage, SYNTAX score, level of ALT, AST, Scr, eGFR, and other clinical parameters. Among these variables, increasing age (estimate = 0.0140, *p* < 0.0001), higher dosage (estimate = 0.0173, *p* = 0.0002), higher SYNTAX score (estimate = 0.0059, *p* = 0.0161), higher level of ALT (estimate = 0.0059, *p* = 0.0012), and Scr (estimate = 0.0012, *p* = 0.0008) were independently associated with a higher plasma AT-all concentration (Table 1).

In stage II, of the patients with RST therapy, 11 (1.74%) were taking 5 mg RST, 549 (86.73%) were 10 mg RST, 67 (10.58%) were 20 mg RST and 6 (0.95%) were 40 mg RST. Multiple linear regression analysis showed that plasma RST concentration was lower in patients with lower level of AST (estimate = −0.0059, *p* = 0.0286) and using angiotensin converting enzyme inhibitors (estimate = −0.3584, *p* = 0.0320) (Table 2).

**Table 2:** Patient characteristics and their effects on plasma concentration of RST.

**Characteristics**		**Value *N* (%) or mean ±*SD***	**Plasma RST concentration, ng/mL**				
				**Univariate analysis**		**Multivariate analysis**	
			**mean ±*SD***	**Estimate**	***p*-value**	**Estimate**	***p*-value**
**DEMOGRAPHIC DATA**							
Total number		635	3.29 ± 3.57				
Age (years)		62.07 ± 10.50		0.0044	0.5810		
Sex	Female	164 (25.83)	3.67 ± 3.72	−0.2438	0.1992		
	Male	471 (74.17)	3.16 ± 3.51				
Dosage (mg)	5	11 (1.74)	2.79 ± 3.64	−0.0052	0.7932		
	10	549 (86.73)	3.19 ± 3.42				
	20	67 (10.58)	4.32 ± 4.57				
	40	6 (0.95)	2.40 ± 3.53				
SYNTAX score		14.19 ± 12.49		−0.0062	0.3671		
**MEDICAL HISTORY**							
Arrhythmia	No	584 (91.97)	3.20 ± 3.44	0.0445	0.8844		
	Yes	51 (8.03)	4.32 ± 4.73				
Diabetes	No	503 (79.21)	3.11 ± 3.33	0.2358	0.2498		
	Yes	132 (20.79)	3.97 ± 4.32				
Heart failure	No	591 (93.07)	3.14 ± 3.29	0.0189	0.9539		
	Yes	44 (6.93)	5.34 ± 5.90				
Hypertension	No	356 (56.06)	3.21 ± 3.50	0.0603	0.7189		
	Yes	279 (43.94)	3.40 ± 3.67				
Hyperlipidemia	No	566 (89.13)	3.27 ± 3.57	0.1139	0.6699		
	Yes	69 (10.87)	3.45 ± 3.63				
**BIOCHEMICAL MEASUREMENTS**							
ALT, U/L		29.27 ± 18.80		0.0021	0.6281		
AST, U/L		31.19 ± 30.87		−0.0064	0.0180	−0.0059	0.0286
Scr, μmol/L		88.66 ± 40.28		0.0007	0.7517		
eGFR, mL/min/1.73 m^2^		98.55 ± 83.96		−0.0003	0.7818		
CK, U/L		146.90 ± 321.49		−0.0004	0.1253		
CKMB, U/L		7.99 ± 11.10		−0.0100	0.1901		
CHOL, mmol/L		4.53 ± 1.45		−0.1029	0.0753		
LDLC, mmol/L		2.77 ± 1.13		−0.2219	0.0029		
HDLC, mmol/L		1.00 ± 0.26		−0.3090	0.3473		
TRIG, mmol/L		1.67 ± 1.24		0.0994	0.1407		
GLUC, mmol/L		7.04 ± 3.20		−0.0251	0.3342		
Lpa, mg/L		275.42 ± 292.45		−0.0006	0.0658		
APOA, g/L		1.06 ± 0.27		0.1348	0.6887		
**MEDICATION**							
β-blockers	No	86 (13.54)	3.71 ± 4.11	−0.3353	0.1675		
	Yes	549 (86.46)	3.22 ± 3.48				
ACEIs	No	284 (44.72)	3.54 ± 3.59	−0.3823	0.0220	−0.3584	0.0320
	Yes	351 (55.28)	3.09 ± 3.55				
CCBs	No	455 (71.65)	3.35 ± 3.70	−0.0735	0.6904		
	Yes	180 (28.35)	3.15 ± 3.24				
PPIs	No	288 (45.35)	3.04 ± 3.18	0.1108	0.5070		
	Yes	347 (54.65)	3.49 ± 3.86				

*Estimates were calculated by applying a linear regression model.Variables with P < 0.05 were entered into the multivariate model, and only variables with P < 0.05 were retained in the model*.

*RST, rosuvastatin; other abbreviations as in Table 1*.

### Effects of baseline characteristics and plasma exposure of AT and metabolites on CI-AKI

**Figure 2:**
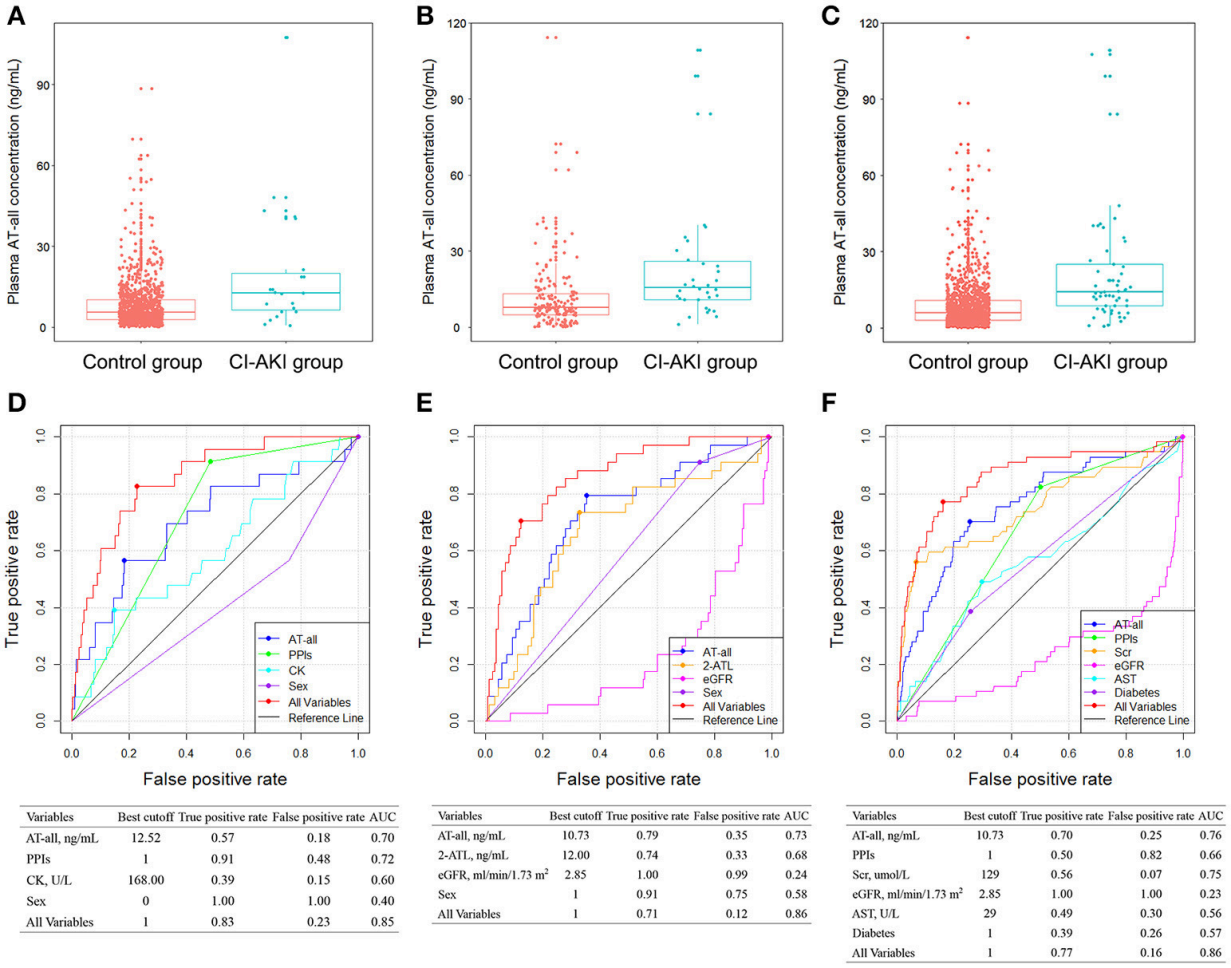
Comparison of plasma AT-all concentration between control group and CI-AKI group in patients without CKD **(A)**, in patients with CKD **(B)**, and in all patients **(C)** in stage I. ROC analyses of variables for predicting CI-AKI in patients without CKD **(D**), in patients with CKD **(E)**, and in all patients **(F)** in stage I. 2-ATL, 2-hydroxy atorvastatin lactone; AST, aspartate aminotransferase; CK, creatine kinase; eGFR, estimated glomerular filtration rate; PPIs, proton pump inhibitors; ROC, receiver operating characteristic; Scr, serum creatinine; other abbreviations as in Figure 1.

In patients receiving AT therapy in stage I, the clinical endpoint CI-AKI within 48 h after the surgery occurred in 57 (4.7%) patients. Univariate logistic analysis showed that higher plasma exposure of AT-all, AT and its five metabolites, SYNTAX core, and other factors were associated with a higher risk of CI-AKI (Table 3). Multivariate analysis revealed that high plasma AT-all exposure (OR: 2.265; 95% CI: 1.609–3.187; *p* < 0.0001), diabetes (OR: 1.953; 95% CI: 1.030–3.704; *p* = 0.0403), high level of AST (OR: 1.009; 95% CI: 1.004–1.015; *p* = 0.0013), Scr (OR: 1.003; 95% CI: 1.001–1.006; *p* = 0.0118), eGFR (OR: 0.977; 95% CI: 0.964–0.991; *p* = 0.0017), and use of proton pump inhibitors (PPIs) (OR: 3.979; 95% CI: 1.828–8.659; *p* = 0.0005) were independent risk factors for CI-AKI (Table 3). Plasma AT-all concentration in patients with CI-AKI (22.40 ± 24.63 ng/mL) was 2.6-fold higher than that in controls (8.60 ± 9.65 ng/mL) (Figure 2C).

**Table 3:** Effects of baseline characteristics and plasma concentrations of AT and its metabolites on CI-AKI in stage I.

**Characteristics**		**Without CI-AKI**	**With CI-AKI**	**Univariate analysis**		**Multivariate analysis**	
		***N* (%) or mean ± *SD***	***N* (%) or mean ± *SD***	**OR (95% CI)**	***P*-Value**	**OR (95% CI)**	***P*-Value**
**DEMOGRAPHIC DATA**							
Total number		1162	57				
Age		62.78 ± 10.03	66.39 ± 8.97	1.040 (1.010–1.071)	0.0084		
Sex	Female	284 (24.44)	13 (22.81)	1.095 (0.581–2.062)	0.7792		
	Male	878 (75.56)	44 (77.19)				
Dosage (mg)	10	19 (1.64)	2 (3.51)	1.022 (0.986–1.059)	0.2268		
	20	1013 (87.18)	45 (78.95)				
	40	130 (11.19)	10 (17.54)				
SYNTAX score		13.54 ± 12.13	19.38 ± 12.23	1.036 (1.015–1.057)	0.0006		
**MEDICAL HISTORY**							
PCI	No	399 (34.34)	17 (29.82)	1.230 (0.689–2.198)	0.4837		
	Yes	763 (65.66)	40 (70.18)				
Arrhythmia	No	1053 (90.93)	51 (89.47)	1.180 (0.495–2.814)	0.7092		
	Yes	105 (9.07)	6 (10.53)				
Diabetes	No	860 (74.27)	35 (61.4)	1.814 (1.047–3.142)	0.0336	1.953 (1.030–3.704)	0.0403
	Yes	298 (25.73)	22 (38.6)				
Heart failure	No	1054 (91.02)	43 (75.44)	3.300 (1.747–6.232)	0.0002		
	Yes	104 (8.98)	14 (24.56)				
Hypertension	No	484 (41.76)	15 (26.32)	2.008 (1.101–3.662)	0.0230		
	Yes	675 (58.24)	42 (73.68)				
Hyperlipidemia	No	1022 (88.18)	51 (89.47)	0.878 (0.370–2.083)	0.7673		
	Yes	137 (11.82)	6 (10.53)				
**BIOCHEMICAL MEASUREMENTS**							
ALT, U/L		28.62 ± 16.69	30.17 ± 19.10	1.005 (0.990–1.020)	0.4960		
AST, U/L		30.34 ± 26.79	45.42 ± 70.84	1.008 (1.003–1.012)	0.0018	1.009 (1.004–1.015)	0.0013
Scr, μmol/L		90.37 ± 47.95	225.43 ± 282.21	1.008 (1.005–1.012)	<0.0001	1.003 (1.001–1.006)	0.0118
eGFR, mL/min/1.73 m^2^		93.03 ± 73.90	55.17 ± 35.25	0.958 (0.948–0.968)	<0.0001	0.977 (0.964–0.991)	0.0017
CK, U/L		151.84 ± 353.97	297.94 ± 991.87	1.000 (1.000–1.001)	0.0261		
CKMB, U/L		8.58 ± 11.80	13.46 ± 32.79	1.013 (1.002–1.024)	0.0247		
CHOL, mmol/L		4.33 ± 1.14	4.15 ± 1.03	0.865 (0.672–1.112)	0.2574		
LDLC, mmol/L		2.60 ± 0.95	2.54 ± 0.89	0.925 (0.689–1.240)	0.6012		
HDLC, mmol/L		0.99 ± 0.26	0.92 ± 0.26	0.292 (0.092–0.922)	0.0358		
TRIG, mmol/L		1.59 ± 1.12	1.52 ± 0.76	0.935 (0.702–1.247)	0.6481		
GLUC, mmol/L		6.64 ± 2.59	6.96 ± 2.69	1.043 (0.950–1.144)	0.3780		
Lpa, mg/L		291.99 ± 310.42	356.40 ± 408.46	1.001 (1.000–1.001)	0.1795		
APOA, g/L		1.07 ± 0.29	0.99 ± 0.24	0.343 (0.102–1.154)	0.0839		
CM volume, mL		148.43 ± 68.01	144.86 ± 51.83	0.999 (0.994–1.005)	0.7531		
**MEDICATION**							
β-blockers	No	137 (11.82)	0 (0)	4.752 (0.897–∞)	0.0019		
	Yes	1022 (88.18)	57 (100)				
ACEIs	No	450 (38.83)	17 (29.82)	1.493 (0.836–2.666)	0.1750		
	Yes	709 (61.17)	40 (70.18)				
CCBs	No	818 (70.58)	30 (52.63)	2.159 (1.264–3.687)	0.0048		
	Yes	341 (29.42)	27 (47.37)				
PPIs	No	578 (49.87)	10 (17.54)	4.676 (2.340–9.343)	<0.0001	3.979 (1.828–8.659)	0.0005
	Yes	581 (50.13)	47 (82.46)				
**PLASMA CONCENTRATION**							
AT, ng/mL		3.95 ± 5.39	10.28 ± 12.14	2.224 (1.697–2.915)	<0.0001		
2-AT, ng/mL		3.50 ± 3.39	8.03 ± 9.79	2.300 (1.678–3.153)	<0.0001		
4-AT, ng/mL		1.28 ± 1.94	4.43 ± 5.95	2.503 (1.942–3.226)	<0.0001		
ATL, ng/mL		3.91 ± 6.02	10.11 ± 12.06	1.959 (1.533–2.503)	<0.0001		
2-ATL, ng/mL		8.94 ± 9.70	17.58 ± 14.77	2.033 (1.518–2.724)	<0.0001		
4-ATL, ng/mL		1.66 ± 2.32	4.03 ± 5.24	2.246 (1.721–2.932)	<0.0001		
AT-all, ng/mL		8.60 ± 9.65	22.40 ± 24.63	2.826 (2.075–3.851)	<0.0001	2.265 (1.609–3.187)	<0.0001

*ORs (95% CI) were calculated by applying a logistic regression model. Variables with P < 0.05 were entered into the multivariate model, and only variables with P < 0.05 were retained in the model*.

*2-AT, 2-hydroxy atorvastatin; 2-ATL, 2-hydroxy atorvastatin lactone; 4-AT, 4-hydroxy atorvastatin; 4-ATL, 4-hydroxy atorvastatin lactone; AT, atorvastatin; ATL, atorvastatin lactone; CI, confidence interval; CI-AKI, contrast induced acute kidney injury; CM volume, contrast media volume; OR, odds ratio; PCI, percutaneous coronary intervention; other abbreviations as in Table 1*.

Considering the impact of baseline renal function on CI-AKI, we further divided patients into two subgroups, which were patients without CKD and with CKD. In patients without CKD in stage Ia, CI-AKI occurred in 23 (2.2%) patients. In multivariate logistic model, only plasma AT-all exposure (OR: 2.381 95% CI: 1.459–3.884; *p* = 0.0005), male (OR: 0.327; 95% CI: 0.133–0.805; *p* = 0.0150), CK level (OR: 1.001; 95% CI: 1.000–1.001; *p* = 0.0030), and use of PPIs (OR: 10.128; 95% CI: 2.330–44.028; *p* = 0.0020) were retained in the model implying that they were independent risk factors for CI-AKI (Table S1). Plasma AT-all concentration in patients with CI-AKI (19.88 ± 23.69 ng/mL) was 2.5-fold higher than that in controls (8.08 ± 8.57 ng/mL) (Figure 2A).

In patients with CKD in stage Ib, CI-AKI occurred in 34 (17.3%) patients, which is much higher than that in stage Ia. The association between high plasma AT-all exposure and CI-AKI remained highly significant. Multivariate logistic regression analysis revealed that high plasma AT-all exposure (OR: 5.377 95% CI: 2.403–12.032; *p* < 0.0001) was still an independent risk factors for CI-AKI (Table S2). Plasma AT-all concentration in patients with CI-AKI (24.10 ± 25.45 ng/mL) was 2.0-fold higher than that in controls (11.82 ± 14.29 ng/mL) (Figure 2B).

### Effects of baseline characteristics and plasma exposure of RST and metabolites on CI-AKI

**Figure 3:**
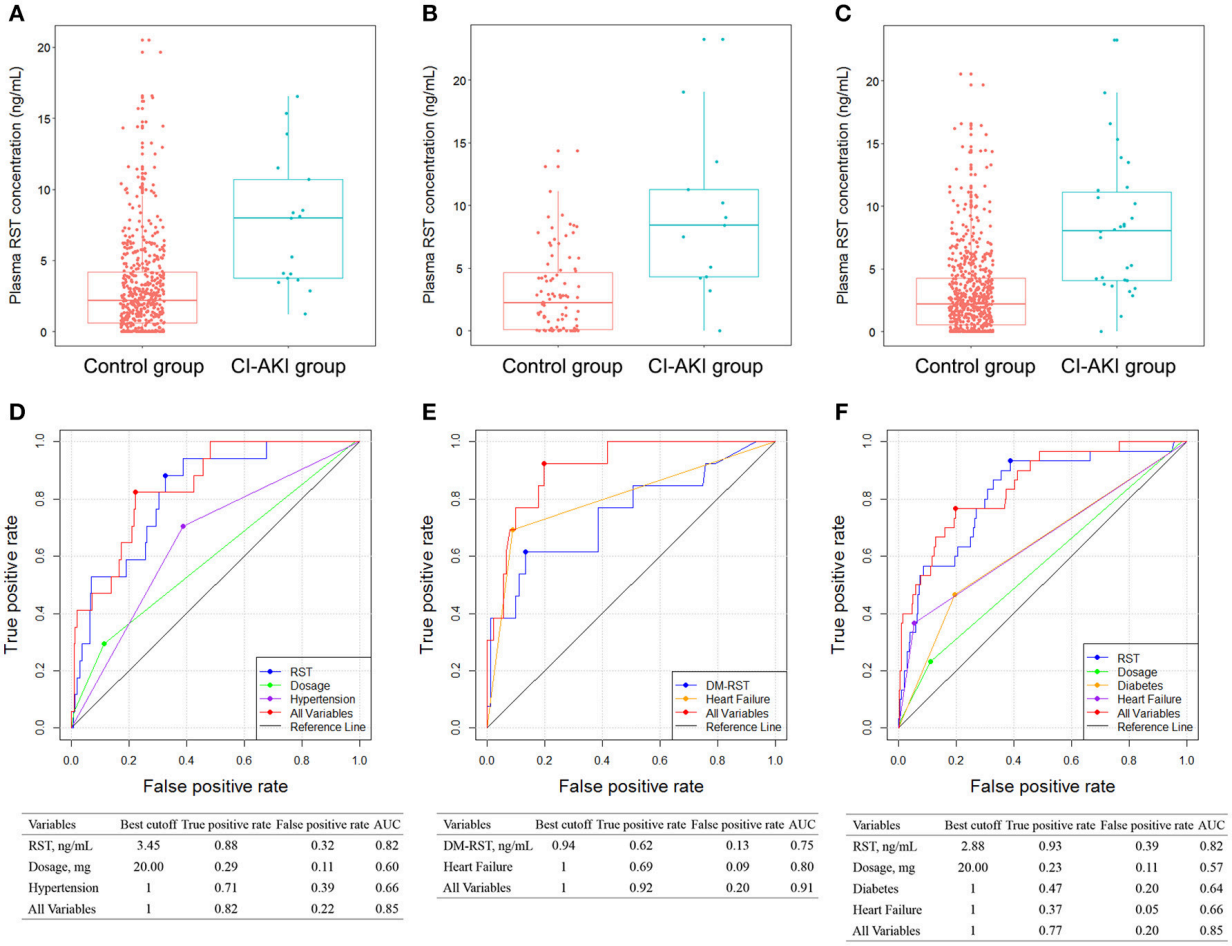
Comparison of plasma RST concentration between control group and CI-AKI group in patients without CKD **(A)**, in patients with CKD **(B)**, and in all patients **(C)** in stage II. ROC analyses of variables for predicting CI-AKI in patients without CKD **(D)**, in patients with CKD **(E)**, and in all patients **(F)** in stage II. DM-RST: N-desmethyl rosuvastatin, RST: rosuvastatin; other abbreviations as in Figures 1, 2.

To further confirm the predictive value of plasma statin concentration for CI-AKI, we assessed the association in patients who received RST treatment in stage II, and the association between high plasma statin exposure and CI-AKI remained highly significant. CI-AKI occurred in 30 (4.7%) patients who received RST treatment. Multivariate logistic regression analysis revealed that patients in the CI-AKI group had higher plasma RST exposure (OR: 2.281; 95% CI: 1.441–3.612; *p* = 0.0004), higher dosage (OR: 1.088; 95% CI: 1.015–1.167; *p* = 0.0175), and were with more diabetes (OR: 2.680; 95% CI: 1.153–6.230; *p* = 0.0220), heart failure (OR: 7.904; 95% CI: 3.032–20.606; *p* < 0.0001) (Table 4). RST plasma concentration in the CI-AKI group (8.28 ± 5.49 ng/mL) was 2.7-fold higher than that in the control group (3.04 ± 3.26 ng/mL) (Figure 3C).

**Table 4:** Effects of baseline characteristics and plasma concentrations of RST and its metabolites on CI-AKI in stage II.

**Characteristics**		**Without CI-AKI**	**With CI-AKI**	**Univariate analysis**		**Multivariate analysis**	
		***N* (%) or mean ±*SD***	***N* (%) or mean ±*SD***	**OR (95% CI)**	***P*-Value**	**OR (95% CI)**	***P*-Value**
**DEMOGRAPHIC DATA**							
Total number		605	30				
Age		61.98 ± 10.53	64.02 ± 9.89	1.020 (0.983–1.058)	0.2995		
Sex	Female	161 (26.61)	3 (10)	3.264 (0.977–10.904)	0.0546		
	Male	444 (73.39)	27 (90)				
Dosage (mg)	5	11 (1.82)	0 (0)	1.065 (1.007–1.127)	0.0285	1.088 (1.015–1.167)	0.0175
	10	528 (87.27)	23 (76.67)				
	20	61 (10.08)	6 (20)				
	40	5 (0.83)	1 (3.33)				
SYNTAX score		14.08 ± 12.57	16.30 ± 10.90	1.014 (0.986–1.042)	0.3440		
**MEDICAL HISTORY**							
PCI	No	269 (44.46)	8 (26.67)	2.202 (0.965–5.023)	0.0608		
	Yes	336 (55.54)	22 (73.33)				
Arrhythmia	No	557 (92.07)	27 (90)	1.290 (0.378–4.406)	0.6848		
	Yes	48 (7.93)	3 (10)				
Diabetes	No	487 (80.5)	16 (53.33)	3.611 (1.714–7.606)	0.0007	2.680 (1.153–6.230)	0.0220
	Yes	118 (19.5)	14 (46.67)				
Heart failure	No	572 (94.55)	19 (63.33)	10.035 (4.414–22.814)	<0.0001	7.904 (3.032–20.606)	<0.0001
	Yes	33 (5.45)	11 (36.67)				
Hypertension	No	347 (57.36)	9 (30)	3.138 (1.414–6.965)	0.0049		
	Yes	258 (42.64)	21 (70)				
Hyperlipidemia	No	539 (89.09)	27 (90)	0.907 (0.268–3.073)	0.8760		
	Yes	66 (10.91)	3 (10)				
**BIOCHEMICAL MEASUREMENTS**							
ALT, U/L		29.31 ± 19.00	28.39 ± 14.23	0.997 (0.977–1.018)	0.7931		
AST, U/L		31.01 ± 30.52	34.78 ± 37.62	1.003 (0.994–1.012)	0.5190		
Scr, μmol/L		87.70 ± 40.27	107.88 ± 36.01	1.007 (1.001–1.013)	0.0148		
eGFR, mL/min/1.73 m^2^		99.77 ± 85.57	74.08 ± 31.24	0.981 (0.968–0.994)	0.0043		
CK, U/L		143.38 ± 311.70	215.29 ± 475.95	1.000 (1.000–1.001)	0.2571		
CKMB, U/L		7.91 ± 10.98	9.65 ± 13.47	1.009 (0.987–1.032)	0.4164		
CHOL, mmol/L		4.54 ± 1.43	4.39 ± 1.78	0.928 (0.699–1.230)	0.6018		
LDLC, mmol/L		2.77 ± 1.11	2.69 ± 1.42	0.931 (0.657–1.318)	0.6853		
HDLC, mmol/L		1.00 ± 0.26	0.99 ± 0.22	0.952 (0.224–4.036)	0.9465		
TRIG, mmol/L		1.67 ± 1.26	1.55 ± 1.02	0.898 (0.610–1.323)	0.5858		
GLUC, mmol/L		7.03 ± 3.20	7.17 ± 3.28	1.013 (0.908–1.130)	0.8163		
Lpa, mg/L		273.94 ± 290.34	301.20 ± 331.66	1.000 (0.999–1.001)	0.6253		
APOA, g/L		1.06 ± 0.28	1.02 ± 0.21	0.501 (0.109–2.308)	0.3752		
CM volume, mL		112.23 ± 60.23	101.09 ± 49.86	0.997 (0.989–1.004)	0.3839		
**MEDICATION**							
β-blockers	No	81 (13.39)	5 (16.67)	0.773 (0.288–2.076)	0.6091		
	Yes	524 (86.61)	25 (83.33)				
ACEIs	No	274 (45.29)	10 (33.33)	1.656 (0.762–3.596)	0.2028		
	Yes	331 (54.71)	20 (66.67)				
CCBs	No	432 (71.4)	23 (76.67)	0.760 (0.320–1.804)	0.5339		
	Yes	173 (28.6)	7 (23.33)				
PPIs	No	277 (45.79)	11 (36.67)	1.458 (0.682–3.117)	0.3301		
	Yes	328 (54.21)	19 (63.33)				
**PLASMA CONCENTRATION**							
RST, ng/mL		3.04 ± 3.26	8.28 ± 5.49	3.139 (1.879–5.245)	<0.0001	2.281 (1.441–3.612)	0.0004
RSTL, ng/mL		0.44 ± 0.53	0.73 ± 0.67	1.498 (1.091–2.058)	0.0125		
DM-RST, ng/mL		0.40 ± 0.54	1.06 ± 1.08	2.610 (1.768–3.853)	<0.0001		

*Variables with P < 0.05 were entered into the multivariate model, and only variables with P < 0.05 were retained in the model*.

*Abbreviations as in Tables 1–3*.

Patients without CKD and with CKD were also analyzed separately. In patients without CKD in stage IIa, CI-AKI occurred in 17 (3.2%) patients. Plasma exposure of RST and DM-RST, dosage, hypertension, level of CK, and CKMB were entered into the multivariate logistic model, and only plasma RST exposure (OR: 3.556; 95% CI: 1.763–7.171; *p* = 0.0004), dosage (OR: 1.083; 95% CI: 1.000–1.173; *p* = 0.0493), and hypertension (OR: 3.492; 95% CI: 1.167–10.450; *p* = 0.0253) were retained in the model implying that they were independent risk factors for CI-AKI (Table S3). CI-AKI patients (7.61 ± 4.66 ng/mL) exhibited 2.5-fold higher plasma RST concentration than controls (3.04 ± 3.26 ng/mL) (Figure 3A).

In patients with CKD in stage IIb, CI-AKI occurred in 13 (12.5%) patients. Higher plasma exposure of RST and DM-RST were associated with a higher risk of CI-AKI. Multivariate analysis revealed that patients in the CI-AKI group had higher plasma DM-RST exposure (OR: 1.935; 95% CI: 1.056–3.547; *p* = 0.0327), and were with more heart failure (OR: 18.817; 95% CI: 4.334–81.701; *p* < 0.0001) (Table S4). CI-AKI patients (9.15 ± 6.51 ng/mL) exhibited 3.0-fold higher plasma RST concentration than controls (3.05 ± 3.28 ng/mL) (Figure 3B).

### Predictive diagnostic power of plasma AT and RST exposure for CI-AKI

To better predict occurrence of CI-AKI, we developed a prognostic classifier combining clinical characteristics and plasma concentrations. In stage I, the AUCs of AT-all, in groups without and with CKD, performed similar characteristics (0.70 and 0.73, respectively) (Figures 2D,E). The AUC of plasma AT-all exposure for all patients in stage I was 0.76. A cutoff of 10.73 ng/mL performed high true positive rate (70%) and low false positive rate (25%) (Figure 2F). Predictive value of use of PPIs, diabetes, level of Scr, AST, and eGFR for CI-AKI, as determined by the ROC curves, were insufficient with AUC varying between 0.23 and 0.75. However, predictive effectiveness of combined variables was substantially increased to 0.86.

AUC for predicting CI-AKI was calculated in an additional independent cohort that receiving RST treatment (stage II study, Figures 3D,E). As shown in Figure 3F, plasma RST exposure was validated in predicting CI-AKI in the stage II cohort (AUC = 0.82). Moreover, the best cutoff of plasma RST exposure was 2.88 ng/mL, and the predictive performance of plasma RST exposure in the validation set yielded high true positive rate (93%) and low false positive rate (39%). After integrating all variable, AUC for the CI-AKI classifier was 0.85.

## Discussion

Statins are one of the most commonly prescribed medications all over the world, rendering it important to determine their potentially effects in CI-AKI. To the best of our knowledge, this is the first study to investigate the risk of CI-AKI in relation to high plasma statin exposure in a prospective study of patients with CAD. Our study provided solid evidence that high plasma statins exposure independently increased the risk of CI-AKI, after adjusting for several potential confounding variables, including demographics, severity of CAD, clinical measurements, prevalent comorbidities, and concomitant use of medications. These findings have important implications for the management of statins therapy in patients undergoing CAG.

Additionally, this study identified that the use of PPIs was significantly and independently associated with increased risk of CI-AKI, and should be administered carefully. These findings are also in agreement with studies reporting (Arora et al., 2016; Lazarus et al., 2016; Moledina and Perazella, 2016) that PPIs are emerging as an important contributing cause to CKD. Clinicians should closely monitor patients taking PPIs by urinalysis and renal function tests to recognize any renal insufficiency in time.

With the increasing role of contrast media in diagnostic and interventional procedures, especially in the field of cardiology, the prevalence of CI-AKI is expected to rise. Pathophysiology of CI-AKI is not exactly understood, and multiple causes, including acute tubular necrosis from poor perfusion, nephrotoxicity from contrast media, use of nephrotoxic medications, cholesterol embolization, procedure-related factors, or a combination of these, may be involved (Kooiman et al., 2015). The prevention of CI-AKI can decrease mortality, morbidity, therapeutic costs and hospital stays. The role of various drugs in prevention of CI-AKI (such as statins) is still controversial and warrants future studies. Some researchers have reported no association between statin use and CI-AKI, whereas others have found that statin use is protective against the incidence of CI-AKI, or that it is associated with an increased risk of CI-AKI. In 2012, Li et al. (2012) performed a meta-analysis including 7 studies and 1,399 patients that investigated the potential benefit of short-term high-dose statins in the prevention of CI-AKI. The results of this study demonstrated that a significant improvement in the incidence of CI-AKI. On the contrary, it has been reported that a short-term administration of high doses of atorvastatin before and after contrast exposure does not decrease the incidence of contrast-induced nephropathy in patients with pre-existing CKD (Toso et al., 2010). One potential factor contributing to this discrepancy is the presence of marked clinical and statistical heterogeneity between studies.

Our results confirm the findings from previous retrospective observational analysis (Dormuth et al., 2013) and retrospective cohort study (Chung et al., 2013). The former discovered that in patients with non-chronic kidney disease, current users of high-potency statins were 34% more likely to be hospitalized with AKI within 120 days after starting treatment (Dormuth et al., 2013). The latter found that statins with high cholesterol-lowering efficacy might increase the risk for developing severe renal failure (Chung et al., 2013). The biological mechanism of statins on kidney injury remained not fully investigated. The higher risk of CI-AKI in patients with high potency statins treatment may be related to an increased risk of proteinuria or rhabdomyolysis. Our results was also supported by an experimental study that AT with a dose of 150 mg/kg/day for 7 days was nephrotoxic for rats, whereas lower doses at 10 or 50 mg/kg/day for 7 days were not accompanied by renal injury (Nasri et al., 2016), suggesting that administration of statins in high doses may itself be directed to renal tubular cell injury.

CI-AKI is likely to remain a significant challenge for cardiologists in the future because the prevalence of comorbid conditions with aging among patients with CKD. It is more frequent in elderly patients with renal insufficiency. Some authors agreed that when renal function is normal, there is no risk of CI-AKI (Andreucci et al., 2017). Therefore, we further divided patients into two subgroups according to baseline renal function, and discovered that patients with CKD had higher incidence than that in patients without CKD. The association between high plasma statin exposure and CI-AKI still remained highly significant in both subgroups.

Several limitations of this study need to be mentioned. Firstly, it was a single-center study and the sample size was relatively small. To address this defect, we used other patient populations that receiving other statin treatment to validate and confirm the predictive value of plasma statin and its metabolites concentrations for CI-AKI. Secondly, we detected the statin concentration at 10–12 h after dose instead of whole profile. However, in the clinical setting, it is easier to monitor steady statin concentration. Additional large-scale, multicenter clinical trial of statin efficacy in CAD patients is thus warranted.

## Conclusion

Our study demonstrated that high plasma exposure of AT and its metabolites could significantly increase the risk of CI-AKI, which was further validated and confirmed in patients receiving RST treatment. Thus, statins should be used with greater caution in patients with CAD undergoing CAG, and plasma levels of statin and metabolites should be monitored to reduce the occurrence of CI-AKI.

## Author contributions

LC, XB, HL and HW performed experiment, performed data analysis, and wrote the manuscript; QZ, ShaZ and YiL participated in data analysis; YoL, GH participated in patient recruitment; QL, JC and BZ revised manuscript; ShiZ, MH and QG designed the study and revised manuscript. All authors reviewed and approved the final manuscript.

### Conflict of interest statement

The authors declare that the research was conducted in the absence of any commercial or financial relationships that could be construed as a potential conflict of interest.
