# Supplementary material for: High Plasma Exposure of Statins Associated With Increased Risk of Contrast-Induced Acute Kidney Injury in Chinese Patients With Coronary Artery Disease
Source: Front Pharmacol. 2018 Apr 30;9:427. doi: 10.3389/fphar.2018.00427 (PMC5936793; doi:10.3389/fphar.2018.00427)
Supplement: Supplementary file 1 [file Table_1.DOCX]

**Table S1**. Effects of baseline characteristics and plasma concentrations of AT and its metabolites on CI-AKI in stage Ia. ORs (95% CI) were calculated by applying a logistic regression model.

| **Characteristics** |  | **Without CI-AKI** | **With CI-AKI** | **Univariate Analysis** | | **Multivariate Analysis** | |
| --- | --- | --- | --- | --- | --- | --- | --- |
|  |  | **N (%) or mean ± SD** | **N (%) or mean ± SD** | **OR (95% CI)** | **P Value** | **OR (95% CI)** | **P Value** |
| **Demographic data** |  |  |  |  |  |  |  |
| Total number |  | 1000 | 23 |  |  |  |  |
| Age |  | 61.73 ± 9.85 | 64.08 ± 8.88 | 1.026 (0.982-1.072) | 0.2591 |  |  |
| Sex | Female | 243 (24.3) | 10 (43.48) | 0.417 (0.181-0.964) | 0.0407 | 0.327 (0.133-0.805) | 0.0150 |
|  | Male | 757 (75.7) | 13 (56.52) |  |  |  |  |
| Dosage (mg) | 10 | 17 (1.7) | 0 (0) | 1.041 (0.991-1.094) | 0.1111 |  |  |
|  | 20 | 870 (87) | 18 (78.26) |  |  |  |  |
|  | 40 | 113 (11.3) | 5 (21.74) |  |  |  |  |
| SYNTAX score |  | 13.00 ± 11.95 | 15.16 ± 11.66 | 1.014 (0.981-1.049) | 0.4016 |  |  |
| **Medical history** |  |  |  |  |  |  |  |
| PCI | No | 354 (35.4) | 8 (34.78) | 1.027 (0.431-2.447) | 0.9514 |  |  |
|  | Yes | 646 (64.6) | 15 (65.22) |  |  |  |  |
| Arrhythmia | No | 916 (91.97) | 21 (91.3) | 1.090 (0.251-4.735) | 0.9079 |  |  |
|  | Yes | 80 (8.03) | 2 (8.7) |  |  |  |  |
| Diabetes | No | 752 (75.5) | 16 (69.57) | 1.349 (0.548-3.317) | 0.5146 |  |  |
|  | Yes | 244 (24.5) | 7 (30.43) |  |  |  |  |
| Heart failure | No | 925 (92.87) | 20 (86.96) | 1.955 (0.567-6.735) | 0.2884 |  |  |
|  | Yes | 71 (7.13) | 3 (13.04) |  |  |  |  |
| Hypertension | No | 443 (44.43) | 7 (30.43) | 1.828 (0.745-4.482) | 0.1876 |  |  |
|  | Yes | 554 (55.57) | 16 (69.57) |  |  |  |  |
| Hyperlipidemia | No | 878 (88.06) | 22 (95.65) | 0.335 (0.045-2.511) | 0.2875 |  |  |
|  | Yes | 119 (11.94) | 1 (4.35) |  |  |  |  |
| **Biochemical measurements** | |  |  |  |  |  |  |
| ALT, U/L |  | 28.97 ± 16.70 | 36.46 ± 22.08 | 1.020 (1.001-1.039) | 0.0388 |  |  |
| AST, U/L |  | 30.34 ± 27.50 | 62.90 ± 103.93 | 1.010 (1.005-1.015) | 0.0003 |  |  |
| Scr, umol/L |  | 79.90 ± 17.00 | 79.40 ± 16.47 | 0.998 (0.974-1.023) | 0.8913 |  |  |
| eGFR, ml/min/1.73 m^2^ |  | 100.54 ± 76.80 | 90.75 ± 22.76 | 0.994 (0.976-1.012) | 0.5049 |  |  |
| CK, U/L |  | 149.81 ± 303.00 | 550.06 ± 1526.80 | 1.001 (1.000-1.001) | 0.0015 | 1.001 (1.000-1.001) | 0.0030 |
| CKMB, U/L |  | 8.44 ± 10.96 | 21.59 ± 49.75 | 1.020 (1.007-1.033) | 0.0020 |  |  |
| CHOL, mmol/L |  | 4.34 ± 1.13 | 4.33 ± 0.92 | 0.989 (0.684-1.430) | 0.9520 |  |  |
| LDLC, mmol/L |  | 2.62 ± 0.95 | 2.61 ± 0.75 | 0.985 (0.634-1.530) | 0.9457 |  |  |
| HDLC, mmol/L |  | 1.00 ± 0.27 | 0.97 ± 0.25 | 0.670 (0.131-3.434) | 0.6306 |  |  |
| TRIG, mmol/L |  | 1.55 ± 1.09 | 1.64 ± 0.81 | 1.059 (0.783-1.431) | 0.7096 |  |  |
| GLUC, mmol/L |  | 6.61 ± 2.57 | 6.42 ± 1.67 | 0.969 (0.812-1.156) | 0.7239 |  |  |
| Lpa, mg/L |  | 281.96 ± 302.43 | 238.66 ± 259.87 | 0.999 (0.998-1.001) | 0.5372 |  |  |
| APOA, g/L |  | 1.08 ± 0.30 | 1.03 ± 0.20 | 0.574 (0.105-3.145) | 0.5221 |  |  |
| CM volume, mL |  | 149.56 ± 69.55 | 132.86 ± 47.62 | 0.994 (0.983-1.006) | 0.3270 |  |  |
| **Medication** |  |  |  |  |  |  |  |
| β-blockers | No | 127 (12.74) | 0 (0) | 4.752 (1.036-∞) | 0.0906 |  |  |
|  | Yes | 870 (87.26) | 23 (100) |  |  |  |  |
| ACEIs | No | 385 (38.62) | 9 (39.13) | 0.979 (0.419-2.283) | 0.9599 |  |  |
|  | Yes | 612 (61.38) | 14 (60.87) |  |  |  |  |
| CCBs | No | 724 (72.62) | 14 (60.87) | 1.705 (0.730-3.984) | 0.2180 |  |  |
|  | Yes | 273 (27.38) | 9 (39.13) |  |  |  |  |
| PPIs | No | 514 (51.55) | 2 (8.7) | 11.174 (2.606-47.908) | 0.0012 | 10.128 (2.330-44.028) | 0.0020 |
|  | Yes | 483 (48.45) | 21 (91.3) |  |  |  |  |
| **Plasma concentration** | |  |  |  |  |  |  |
| AT, ng/mL |  | 3.72 ± 4.89 | 10.30 ± 13.21 | 2.119 (1.401-3.206) | 0.0004 |  |  |
| 2-AT, ng/mL |  | 3.37 ± 3.19 | 6.91 ± 6.96 | 2.090 (1.275-3.424) | 0.0034 |  |  |
| 4-AT, ng/mL |  | 1.12 ± 1.48 | 2.95 ± 4.71 | 1.997 (1.341-2.974) | 0.0007 |  |  |
| ATL, ng/mL |  | 3.64 ± 5.75 | 9.62 ± 14.58 | 1.786 (1.237-2.578) | 0.0020 |  |  |
| 2-ATL, ng/mL |  | 8.43 ± 8.98 | 15.48 ± 14.58 | 1.875 (1.199-2.931) | 0.0058 |  |  |
| 4-ATL, ng/mL |  | 1.52 ± 2.03 | 2.45 ± 2.56 | 1.693 (1.137-2.521) | 0.0095 |  |  |
| AT-all, ng/mL |  | 8.08 ± 8.57 | 19.88 ± 23.69 | 2.359 (1.472-3.782) | 0.0004 | 2.381 (1.459-3.884) | 0.0005 |
| Variables with P < 0.05 were entered into the multivariate model, and only variables with P < 0.05 were retained in the model. | | | | | | | |
| 2-AT = 2-hydroxy atorvastatin; 2-ATL = 2-hydroxy atorvastatin lactone; 4-AT = 4-hydroxy atorvastatin; 4-ATL = 4-hydroxy atorvastatin lactone; ACEIs = angiotensin converting enzyme inhibitors; ALT = alanine aminotransferase; APOA = apolipoprotein a; AST = aspartate aminotransferase; AT = atorvastatin; ATL = atorvastatin lactone; CCBs = calcium channel blockers; CHOL = cholesterol; CI = confidence interval; CI-AKI = contrast induced acute kidney injury; CK = creatine kinase; CKMB = creatine kinase MB; CM volume = contrast media volume; eGFR = estimated glomerular filtration rate; GLUC = glucose; HDLC = high-density lipoprotein cholesterol; LDLC = low-density lipoprotein cholesterol; Lpa = lipoprotein (a); OR =odds ratio; PCI = percutaneous coronary intervention; PPIs = proton pump inhibitors; Scr = serum creatinine; SD = standard deviation; SYNTAX score = Synergy between percutaneous coronary intervention with TAXUS and Cardiac Surgery score; TRIG = triglyceride. | | | | | | | |
|  |  |  |  |  |  |  |  |
|  |  |  |  |  |  |  |  |
|  |  |  |  |  |  |  |  |
|  |  |  |  |  |  |  |  |
|  |  |  |  |  |  |  |  |
