# Supplementary material for: High Plasma Exposure of Statins Associated With Increased Risk of Contrast-Induced Acute Kidney Injury in Chinese Patients With Coronary Artery Disease
Source: Front Pharmacol. 2018 Apr 30;9:427. doi: 10.3389/fphar.2018.00427 (PMC5936793; doi:10.3389/fphar.2018.00427)
Supplement: Supplementary file 2 [file Table_2.DOCX]

**Table S2**. Effects of baseline characteristics and plasma concentrations of AT and its metabolites on CI-AKI in stage Ib. ORs (95% CI) were calculated by applying a logistic regression model.

| **Characteristics** |  | **Without CI-AKI** | **With CI-AKI** | **Univariate Analysis** | | **Multivariate Analysis** | |
| --- | --- | --- | --- | --- | --- | --- | --- |
|  |  | **N (%) or mean ± SD** | **N (%) or mean ± SD** | **OR (95% CI)** | **P Value** | **OR (95% CI)** | **P Value** |
| **Demographic data** |  |  |  |  |  |  |  |
| Total number |  | 162 | 34 |  |  |  |  |
| Age |  | 69.27 ± 8.54 | 67.96 ± 8.81 | 0.983 (0.943-1.025) | 0.4172 |  |  |
| Sex | Female | 41 (25.31) | 3 (8.82) | 3.501 (1.016-12.062) | 0.0471 | 8.041 (1.778-36.359) | 0.0068 |
|  | Male | 121 (74.69) | 31 (91.18) |  |  |  |  |
| Dosage (mg) | 10 | 2 (1.23) | 2 (5.88) | 1.009 (0.955-1.065) | 0.7597 |  |  |
|  | 20 | 143 (88.27) | 27 (79.41) |  |  |  |  |
|  | 40 | 17 (10.49) | 5 (14.71) |  |  |  |  |
| SYNTAX score |  | 16.92 ± 12.69 | 22.20 ± 11.96 | 1.033 (1.003-1.064) | 0.0321 |  |  |
| **Medical history** |  |  |  |  |  |  |  |
| PCI | No | 45 (27.78) | 9 (26.47) | 1.068 (0.463-2.465) | 0.8768 |  |  |
|  | Yes | 117 (72.22) | 25 (73.53) |  |  |  |  |
| Arrhythmia | No | 137 (84.57) | 30 (88.24) | 0.731 (0.237-2.255) | 0.5855 |  |  |
|  | Yes | 25 (15.43) | 4 (11.76) |  |  |  |  |
| Diabetes | No | 108 (66.67) | 19 (55.88) | 1.579 (0.745-3.349) | 0.2335 |  |  |
|  | Yes | 54 (33.33) | 15 (44.12) |  |  |  |  |
| Heart failure | No | 129 (79.63) | 23 (67.65) | 1.870 (0.828-4.219) | 0.1319 |  |  |
|  | Yes | 33 (20.37) | 11 (32.35) |  |  |  |  |
| Hypertension | No | 41 (25.31) | 8 (23.53) | 1.101 (0.462-2.623) | 0.8276 |  |  |
|  | Yes | 121 (74.69) | 26 (76.47) |  |  |  |  |
| Hyperlipidemia | No | 144 (88.89) | 29 (85.29) | 1.380 (0.474-4.014) | 0.5549 |  |  |
|  | Yes | 18 (11.11) | 5 (14.71) |  |  |  |  |
| **Biochemical measurements** | |  |  |  |  |  |  |
| ALT, U/L |  | 26.47 ± 16.50 | 25.91 ± 15.75 | 0.998 (0.975-1.021) | 0.8569 |  |  |
| AST, U/L |  | 30.30 ± 21.93 | 33.59 ± 30.93 | 1.005 (0.991-1.019) | 0.4646 |  |  |
| Scr, umol/L |  | 156.14 ± 99.45 | 324.24 ± 331.72 | 1.004 (1.002-1.007) | 0.0006 |  |  |
| eGFR, ml/min/1.73 m^2^ |  | 46.11 ± 13.28 | 31.11 ± 16.87 | 0.943 (0.920-0.966) | < 0.0001 | 0.939 (0.913-0.965) | < 0.0001 |
| CK, U/L |  | 164.31 ± 577.03 | 122.22 ± 103.69 | 1.000 (0.998-1.001) | 0.6979 |  |  |
| CKMB, U/L |  | 9.39 ± 16.05 | 7.61 ± 5.68 | 0.988 (0.950-1.027) | 0.5476 |  |  |
| CHOL, mmol/L |  | 4.26 ± 1.17 | 4.03 ± 1.10 | 0.833 (0.585-1.185) | 0.3089 |  |  |
| LDLC, mmol/L |  | 2.48 ± 0.93 | 2.49 ± 0.99 | 1.003 (0.671-1.498) | 0.9885 |  |  |
| HDLC, mmol/L |  | 0.95 ± 0.25 | 0.88 ± 0.27 | 0.296 (0.058-1.515) | 0.1439 |  |  |
| TRIG, mmol/L |  | 1.78 ± 1.30 | 1.43 ± 0.71 | 0.699 (0.430-1.136) | 0.1485 |  |  |
| GLUC, mmol/L |  | 6.85 ± 2.70 | 7.32 ± 3.18 | 1.057 (0.934-1.196) | 0.3809 |  |  |
| Lpa, mg/L |  | 353.73 ± 350.72 | 439.24 ± 474.08 | 1.001 (1.000-1.002) | 0.2832 |  |  |
| APOA, g/L |  | 1.00 ± 0.23 | 0.96 ± 0.26 | 0.469 (0.072-3.054) | 0.4285 |  |  |
| CM volume, mL |  | 142.11 ± 58.61 | 152.17 ± 53.93 | 1.003 (0.995-1.011) | 0.4551 |  |  |
| **Medication** |  |  |  |  |  |  |  |
| β-blockers | No | 10 (6.17) | 0 (0) | 3.063 (0.6100-∞) | 0.2831 |  |  |
|  | Yes | 152 (93.83) | 34 (100) |  |  |  |  |
| ACEIs | No | 65 (40.12) | 8 (23.53) | 2.178 (0.929-5.108) | 0.0735 |  |  |
|  | Yes | 97 (59.88) | 26 (76.47) |  |  |  |  |
| CCBs | No | 94 (58.02) | 16 (47.06) | 1.555 (0.740-3.267) | 0.2436 |  |  |
|  | Yes | 68 (41.98) | 18 (52.94) |  |  |  |  |
| PPIs | No | 64 (39.51) | 8 (23.53) | 2.122 (0.905-4.979) | 0.0837 |  |  |
|  | Yes | 98 (60.49) | 26 (76.47) |  |  |  |  |
| **Plasma concentration** | |  |  |  |  |  |  |
| AT, ng/mL |  | 5.42 ± 7.65 | 10.27 ± 11.57 | 1.774 (1.228-2.563) | 0.0023 |  |  |
| 2-AT, ng/mL |  | 4.35 ± 4.39 | 8.79 ± 11.35 | 1.841 (1.234-2.747) | 0.0028 |  |  |
| 4-AT, ng/mL |  | 2.24 ± 3.51 | 5.44 ± 6.53 | 1.994 (1.412-2.815) | < 0.0001 |  |  |
| ATL, ng/mL |  | 5.54 ± 7.28 | 10.45 ± 10.23 | 1.591 (1.132-2.235) | 0.0075 |  |  |
| 2-ATL, ng/mL |  | 12.11 ± 12.92 | 19.01 ± 14.94 | 1.578 (1.086-2.292) | 0.0167 | 0.416 (0.215-0.807) | 0.0095 |
| 4-ATL, ng/mL |  | 2.49 ± 3.55 | 5.10 ± 6.27 | 1.973 (1.357-2.870) | 0.0004 |  |  |
| AT-all, ng/mL |  | 11.82 ± 14.29 | 24.10 ± 25.45 | 2.310 (1.491-3.579) | 0.0002 | 5.377 (2.403-12.032) | < 0.0001 |
| Variables with P < 0.05 were entered into the multivariate model, and only variables with P < 0.05 were retained in the model. | | | | | | | |
| Abbreviations as in **Table S1**. | | | | | | | |
