# Supplementary material for: High Plasma Exposure of Statins Associated With Increased Risk of Contrast-Induced Acute Kidney Injury in Chinese Patients With Coronary Artery Disease
Source: Front Pharmacol. 2018 Apr 30;9:427. doi: 10.3389/fphar.2018.00427 (PMC5936793; doi:10.3389/fphar.2018.00427)
Supplement: Supplementary file 3 [file Table_3.DOCX]

**Table S3**. Effects of baseline characteristics and plasma concentrations of RST and its metabolites on CI-AKI in stage IIa. ORs (95% CI) were calculated by applying a logistic regression model.

| **Characteristics** |  | **Without CI-AKI** | **With CI-AKI** | **Univariate Analysis** | | **Multivariate Analysis** | |
| --- | --- | --- | --- | --- | --- | --- | --- |
|  |  | **N (%) or mean ± SD** | **N (%) or mean ± SD** | **OR (95% CI)** | **P Value** | **OR (95% CI)** | **P Value** |
| **Demographic data** |  |  |  |  |  |  |  |
| Total number |  | 514 | 17 |  |  |  |  |
| Age |  | 61.19 ± 10.64 | 62.44 ± 9.87 | 1.011 (0.965-1.060) | 0.6323 |  |  |
| Sex | Female | 137 (26.65) | 1 (5.88) | 5.813 (0.764-44.245) | 0.0892 |  |  |
|  | Male | 377 (73.35) | 16 (94.12) |  |  |  |  |
| Dosage (mg) | 5 | 7 (1.36) | 0 (0) | 1.079 (1.014-1.149) | 0.0160 | 1.083 (1.000-1.173) | 0.0493 |
|  | 10 | 449 (87.35) | 12 (70.59) |  |  |  |  |
|  | 20 | 53 (10.31) | 4 (23.53) |  |  |  |  |
|  | 40 | 5 (0.97) | 1 (5.88) |  |  |  |  |
| SYNTAX score |  | 13.37 ± 12.41 | 15.76 ± 12.34 | 1.015 (0.978-1.053) | 0.4354 |  |  |
| **Medical history** |  |  |  |  |  |  |  |
| PCI | No | 236 (45.91) | 4 (23.53) | 2.759 (0.888-8.574) | 0.0794 |  |  |
|  | Yes | 278 (54.09) | 13 (76.47) |  |  |  |  |
| Arrhythmia | No | 477 (92.8) | 17 (100) | 0.543 (0.000-2.586) | 0.5745 |  |  |
|  | Yes | 37 (7.2) |  |  |  |  |  |
| Diabetes | No | 422 (82.1) | 13 (76.47) | 1.411 (0.450-4.427) | 0.5547 |  |  |
|  | Yes | 92 (17.9) | 4 (23.53) |  |  |  |  |
| Heart failure | No | 489 (95.14) | 15 (88.24) | 2.608 (0.565-12.034) | 0.2192 |  |  |
|  | Yes | 25 (4.86) | 2 (11.76) |  |  |  |  |
| Hypertension | No | 315 (61.28) | 5 (29.41) | 3.799 (1.319-10.945) | 0.0134 | 3.492 (1.167-10.450) | 0.0253 |
|  | Yes | 199 (38.72) | 12 (70.59) |  |  |  |  |
| Hyperlipidemia | No | 459 (89.3) | 14 (82.35) | 1.788 (0.498-6.419) | 0.3726 |  |  |
|  | Yes | 55 (10.7) | 3 (17.65) |  |  |  |  |
| **Biochemical measurements** | |  |  |  |  |  |  |
| ALT, U/L |  | 29.33 ± 18.55 | 26.09 ± 11.70 | 0.988 (0.957-1.021) | 0.4768 |  |  |
| AST, U/L |  | 29.09 ± 20.42 | 36.54 ± 49.44 | 1.009 (0.996-1.023) | 0.1877 |  |  |
| Scr, umol/L |  | 76.55 ± 18.03 | 82.74 ± 17.53 | 1.021 (0.992-1.051) | 0.1618 |  |  |
| eGFR, ml/min/1.73 m^2^ |  | 109.25 ± 89.21 | 95.10 ± 25.12 | 0.994 (0.976-1.013) | 0.5372 |  |  |
| CK, U/L |  | 124.41 ± 200.81 | 253.70 ± 581.78 | 1.001 (1.000-1.002) | 0.0472 |  |  |
| CKMB, U/L |  | 7.29 ± 6.88 | 11.58 ± 17.64 | 1.033 (1.001-1.067) | 0.0431 |  |  |
| CHOL, mmol/L |  | 4.54 ± 1.47 | 4.50 ± 2.06 | 0.982 (0.702-1.373) | 0.9151 |  |  |
| LDLC, mmol/L |  | 2.76 ± 1.11 | 2.80 ± 1.73 | 1.030 (0.681-1.557) | 0.8887 |  |  |
| HDLC, mmol/L |  | 1.00 ± 0.26 | 1.06 ± 0.19 | 2.254 (0.416-12.204) | 0.3457 |  |  |
| TRIG, mmol/L |  | 1.69 ± 1.32 | 1.67 ± 1.23 | 0.991 (0.679-1.447) | 0.9642 |  |  |
| GLUC, mmol/L |  | 6.81 ± 2.97 | 6.91 ± 2.51 | 1.011 (0.863-1.183) | 0.8959 |  |  |
| Lpa, mg/L |  | 255.50 ± 262.33 | 255.64 ± 305.73 | 1.000 (0.998-1.002) | 0.9983 |  |  |
| APOA, g/L |  | 1.07 ± 0.28 | 1.03 ± 0.17 | 0.565 (0.082-3.904) | 0.5624 |  |  |
| CM volume, mL |  | 110.82 ± 60.69 | 101.07 ± 47.56 | 0.997 (0.988-1.007) | 0.5522 |  |  |
| **Medication** |  |  |  |  |  |  |  |
| β-blockers | No | 73 (14.2) | 1 (5.88) | 2.647 (0.346-20.251) | 0.3485 |  |  |
|  | Yes | 441 (85.8) | 16 (94.12) |  |  |  |  |
| ACEIs | No | 239 (46.5) | 4 (23.53) | 2.824 (0.909-8.777) | 0.0727 |  |  |
|  | Yes | 275 (53.5) | 13 (76.47) |  |  |  |  |
| CCBs | No | 378 (73.54) | 16 (94.12) | 0.174 (0.023-1.322) | 0.0910 |  |  |
|  | Yes | 136 (26.46) | 1 (5.88) |  |  |  |  |
| PPIs | No | 237 (46.11) | 8 (47.06) | 0.962 (0.366-2.534) | 0.9383 |  |  |
|  | Yes | 277 (53.89) | 9 (52.94) |  |  |  |  |
| **Plasma concentration** |  |  |  |  |  |  |  |
| RST, ng/mL |  | 3.04 ± 3.26 | 7.61 ± 4.66 | 3.994 (1.949-8.185) | 0.0002 | 3.556 (1.763-7.171) | 0.0004 |
| RSTL, ng/mL |  | 0.41 ± 0.44 | 0.69 ± 0.72 | 1.495 (0.970-2.305) | 0.0687 |  |  |
| DM-RST, ng/mL |  | 0.40 ± 0.54 | 0.72 ± 0.52 | 2.357 (1.419-3.915) | 0.0009 |  |  |
| Variables with P < 0.05 were entered into the multivariate model, and only variables with P < 0.05 were retained in the model. | | | | | | | |
| DM-RST = N-desmethyl rosuvastatin; RST = rosuvastatin; RSTL = rosuvastatin lactone; other abbreviations as in **Table S1**. | | | | | | | |
