# Supplementary material for: High Plasma Exposure of Statins Associated With Increased Risk of Contrast-Induced Acute Kidney Injury in Chinese Patients With Coronary Artery Disease
Source: Front Pharmacol. 2018 Apr 30;9:427. doi: 10.3389/fphar.2018.00427 (PMC5936793; doi:10.3389/fphar.2018.00427)
Supplement: Supplementary file 4 [file Table_4.DOCX]

**Table S4**. Effects of baseline characteristics and plasma concentrations of RST and its metabolites on CI-AKI in stage IIb. ORs (95% CI) were calculated by applying a logistic regression model.

| **Characteristics** |  | **Without CI-AKI** | **With CI-AKI** | **Univariate Analysis** | | **Multivariable Analysis** | |
| --- | --- | --- | --- | --- | --- | --- | --- |
|  |  | **N (%) or mean ± SD** | **N (%) or mean ± SD** | **OR (95% CI)** | **P Value** | **OR (95% CI)** | **P Value** |
| **Demographic data** |  |  |  |  |  |  |  |
| Total number |  | 91 | 13 |  |  |  |  |
| Age |  | 66.43 ± 8.67 | 66.08 ± 9.92 | 0.996 (0.932-1.063) | 0.8932 |  |  |
| Sex | Female | 24 (26.37) | 2 (15.38) | 1.970 (0.407-9.538) | 0.3994 |  |  |
|  | Male | 67 (73.63) | 11 (84.62) |  |  |  |  |
| Dosage (mg) | 5 | 4 (4.4) | 0 (0) | 1.076 (0.920-1.258) | 0.3615 |  |  |
|  | 10 | 79 (86.81) | 11 (84.62) |  |  |  |  |
|  | 20 | 8 (8.79) | 2 (15.38) |  |  |  |  |
| SYNTAX score |  | 18.11 ± 12.77 | 17.00 ± 9.11 | 0.992 (0.945-1.042) | 0.7604 |  |  |
| **Medical history** |  |  |  |  |  |  |  |
| PCI | No | 33 (36.26) | 4 (30.77) | 1.280 (0.366-4.481) | 0.6993 |  |  |
|  | Yes | 58 (63.74) | 9 (69.23) |  |  |  |  |
| Arrhythmia | No | 80 (87.91) | 10 (76.92) | 2.182 (0.519-9.171) | 0.2869 |  |  |
|  | Yes | 11 (12.09) | 3 (23.08) |  |  |  |  |
| Diabetes | No | 65 (71.43) | 3 (23.08) | 8.333 (2.122-32.730) | 0.0024 |  |  |
|  | Yes | 26 (28.57) | 10 (76.92) |  |  |  |  |
| Heart failure | No | 83 (91.21) | 4 (30.77) | 23.344 (5.853-93.101) | < 0.0001 | 18.817 (4.334-81.701) | < 0.0001 |
|  | Yes | 8 (8.79) | 9 (69.23) |  |  |  |  |
| Hypertension | No | 32 (35.16) | 4 (30.77) | 1.220 (0.348-4.276) | 0.7556 |  |  |
|  | Yes | 59 (64.84) | 9 (69.23) |  |  |  |  |
| Hyperlipidemia | No | 80 (87.91) | 0 (0) | 0.416 (0.000-2.172) | 0.4244 |  |  |
|  | Yes | 11 (12.09) | 13 (100) |  |  |  |  |
| **Biochemical measurements** | |  |  |  |  |  |  |
| ALT, U/L |  | 29.22 ± 21.51 | 31.39 ± 17.03 | 1.005 (0.979-1.031) | 0.7253 |  |  |
| AST, U/L |  | 41.83 ± 61.07 | 32.48 ± 12.26 | 0.995 (0.978-1.013) | 0.6008 |  |  |
| Scr, umol/L |  | 152.13 ± 65.39 | 140.75 ± 25.65 | 0.996 (0.983-1.009) | 0.5409 |  |  |
| eGFR, ml/min/1.73 m^2^ |  | 44.99 ± 11.96 | 46.59 ± 8.48 | 1.013 (0.960-1.069) | 0.6416 |  |  |
| CK, U/L |  | 253.27 ± 645.19 | 165.07 ± 302.21 | 1.000 (0.998-1.001) | 0.6397 |  |  |
| CKMB, U/L |  | 11.50 ± 23.12 | 7.12 ± 3.33 | 0.978 (0.908-1.053) | 0.5527 |  |  |
| CHOL, mmol/L |  | 4.54 ± 1.21 | 4.26 ± 1.42 | 0.819 (0.487-1.378) | 0.4525 |  |  |
| LDLC, mmol/L |  | 2.82 ± 1.12 | 2.54 ± 0.93 | 0.758 (0.409-1.406) | 0.3800 |  |  |
| HDLC, mmol/L |  | 0.96 ± 0.27 | 0.90 ± 0.23 | 0.391 (0.031-4.924) | 0.4671 |  |  |
| TRIG, mmol/L |  | 1.58 ± 0.80 | 1.38 ± 0.66 | 0.668 (0.272-1.644) | 0.3802 |  |  |
| GLUC, mmol/L |  | 8.27 ± 4.09 | 7.51 ± 4.16 | 0.948 (0.801-1.122) | 0.5334 |  |  |
| Lpa, mg/L |  | 381.36 ± 403.67 | 365.75 ± 369.17 | 1.000 (0.998-1.001) | 0.8989 |  |  |
| APOA, g/L |  | 1.01 ± 0.25 | 0.99 ± 0.26 | 0.766 (0.061-9.692) | 0.8368 |  |  |
| CM volume, mL |  | 120.56 ± 57.22 | 101.11 ± 56.22 | 0.993 (0.979-1.007) | 0.3397 |  |  |
| **Medication** |  |  |  |  |  |  |  |
| β-blockers | No | 8 (8.79) | 4 (30.77) | 0.217 (0.054-0.865) | 0.0303 |  |  |
|  | Yes | 83 (91.21) | 9 (69.23) |  |  |  |  |
| ACEIs | No | 35 (38.46) | 6 (46.15) | 0.729 (0.226-2.348) | 0.5965 |  |  |
|  | Yes | 56 (61.54) | 7 (53.85) |  |  |  |  |
| CCBs | No | 54 (59.34) | 7 (53.85) | 1.251 (0.389-4.022) | 0.7071 |  |  |
|  | Yes | 37 (40.66) | 6 (46.15) |  |  |  |  |
| PPIs | No | 40 (43.96) | 3 (23.08) | 2.614 (0.674-10.135) | 0.1645 |  |  |
|  | Yes | 51 (56.04) | 10 (76.92) |  |  |  |  |
| **Plasma concentration** |  |  |  |  |  |  |  |
| RST, ng/mL |  | 3.05 ± 3.28 | 9.15 ± 6.51 | 2.037 (1.097-3.780) | 0.0241 |  |  |
| RSTL, ng/mL |  | 0.62 ± 0.84 | 0.79 ± 0.62 | 1.328 (0.871-2.025) | 0.1878 |  |  |
| DM-RST, ng/mL |  | 0.45 ± 0.52 | 1.51 ± 1.43 | 2.459 (1.352-4.473) | 0.0032 | 1.935 (1.056-3.547) | 0.0327 |
| Variables with P < 0.05 were entered into the multivariate model, and only variables with P < 0.05 were retained in the model. | | | | | | | |
| Abbreviations as in **Tables S1** and **S3**. | | | | | | | |
